# Exploring accidental virulence

**DOI:** 10.7554/eLife.94556

**Published:** 2024-01-03

**Authors:** Daniel FQ Smith

**Affiliations:** 1 W. Harry Feinstone Department of Microbiology and Immunology, The Johns Hopkins Bloomberg School of Public Health Baltimore United States

**Keywords:** yeast, experimental evolution, virulence, adherence, biofilm, FLO11, *S. cerevisiae*

## Abstract

Experimentally evolving yeast to adhere better to plastic led to adaptations that increased their ability to cause an infection.

**Related research article** Ekdahl LI, Salcedo JA, Dungan MM, Mason DV, Myagmarsuren D, Murphy HA. 2023. Selection on plastic adherence leads to hyper-multicellular strains and incidental virulence in the budding yeast. *eLife* **12**:e81056. doi: 10.7554/eLife.81056.

Not all microbes, such as bacteria and fungi, display virulent properties that allow them to infect host cells and cause disease. While some disease-causing pathogens need a host in order to replicate and survive, some can also live freely in the environment. However, how these pathogens go from innocuously living outside of a host to being able to cause disease within one remains an open question.

One leading theory suggests that these are ‘accidental’ pathogens. According to this ‘accidental virulence theory’, environmental factors cause microbes to gain properties that allow them to survive without a host. While these microbes can exist independently, the adaptations can also make them better equipped to survive, replicate and cause disease if they happen to encounter a potential host (Casadevall and Pirofski, 2007; Figure 1A). For example, microbes that have adapted to living in acidic environments, may also be able to survive in the acidic conditions of animal immune cells, enabling them to infect a host (Hackam et al., 1999). This theory challenges the idea that microbes only acquire the ability to causes disease through exposure and adapting to conditions in a specific host. Now, in eLife, Helen Murphy and colleagues from the College of William and Mary and Vanderbilt University – including Luke Ekdahl and Juliana Salcedo as joint first authors – report evidence supporting the accidental virulence theory using the traditionally non-pathogenic fungus *Saccharomyces cerevisiae* (Figure 1B; Ekdahl et al., 2023).

**Figure 1.**
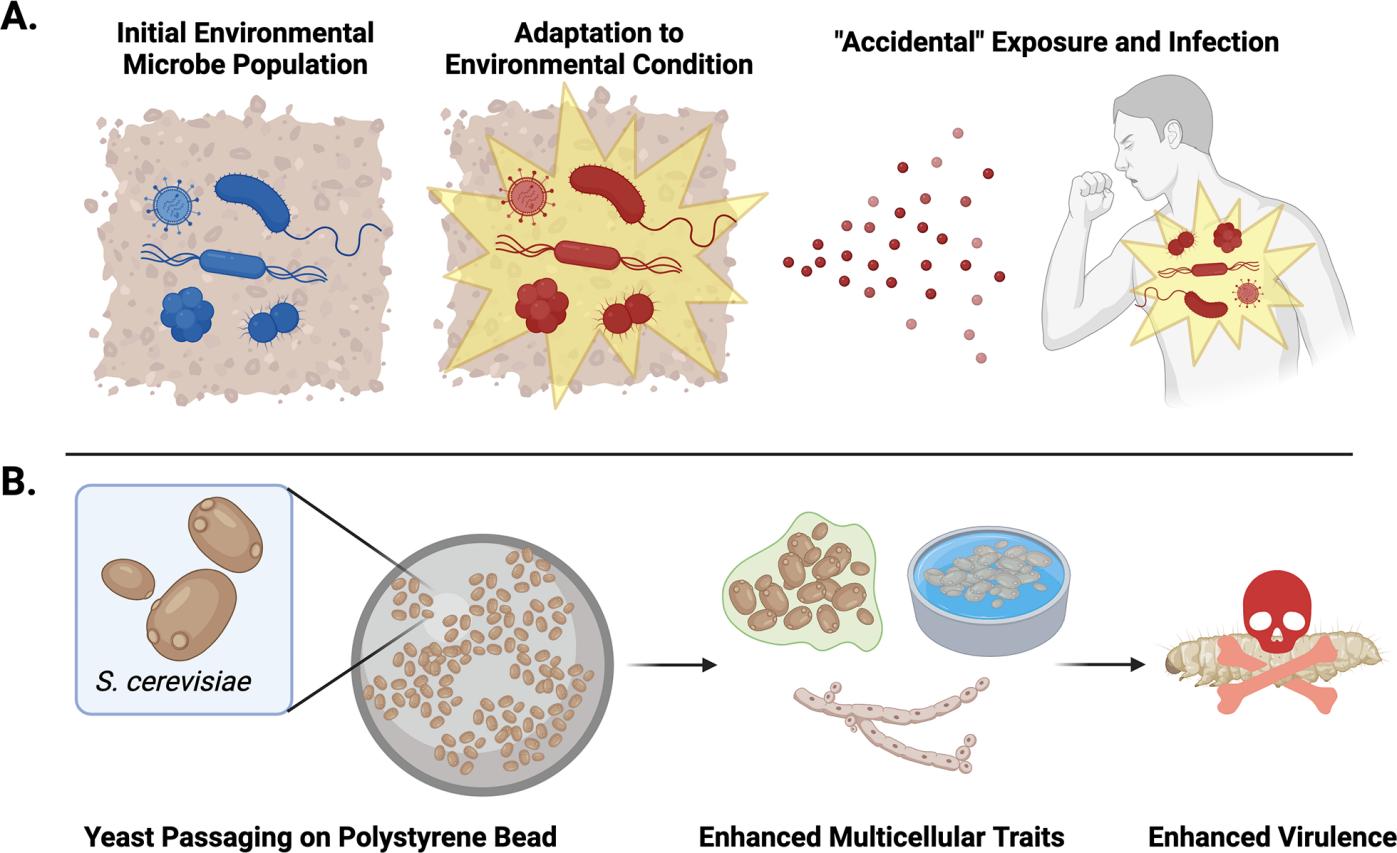
Testing the accidental virulence hypothesis. (**A**) Environmental microbes (blue) adapt to new conditions and stressors in their surroundings. If these adapted microbes (red) come into contact with a potential host, their new adaptations may better equip them to infect and cause disease, despite not having encountered the host previously. (**B**) Ekdahl et al. demonstrated that repeatedly selecting *Saccharomyces cerevisiae* yeast that could grow on polystyrene beads led to the yeast evolving more multicellular traits that resulted in them growing more regularly into a biofilm, flor, or forming pseudohyphae. The enhanced frequency of multicellular traits made the yeast better at infecting wax moth larvae.

Image credit: Figure created using elements from Biorender.com.

Despite *S. cerevisiae,* which is commonly referred to as baker’s yeast, being considered non-pathogenic, there are some instances in which it can infect humans (Enache-Angoulvant and Hennequin, 2005). This makes it an interesting model for studying how adaptations not related directly to virulence may coincidentally allow the yeast to infect a host. Additionally, there are already many readily available tools for teasing apart the mechanism of evolution and increased pathogenic potential in this model organism.

Ekdahl et al. investigated how *S. cerevisiae* characteristics were affected by exposure to plastic – a common component of modern environments due to microplastic pollution. The authors grew two separate *S. cerevisiae* strains on polystyrene beads for 350–400 generations. Throughout this period, only cells that adhered to the beads were transferred to each new bead culture. By doing this, the team created a selection pressure that was specific to the yeast’s interaction with the plastic and not related to how it interacts with the immune system of a potential host. To ensure genetic diversity and mixing of genes amongst the population, fungi that were in their sexual stage were mated during the process.

The experiments showed that selection of plastic-adhering *S. cerevisiae* led to the evolution of the fungus to display multicellular traits more frequently. Typically, *S. cerevisiae* is found in its single-celled oval yeast form. Here, the fungus made more elongated branch-like structures called pseudohyphae, better biofilms (durable structures that help microbial communities adhere to surfaces), and multicellular floating structures called ‘flors’.

All three of these multicellular phenotypes have been associated with increased ability of fungi, including *S. cerevisiae*, to infect mammals by allowing fungal communities to persist and ‘stick’ within the host (Roig et al., 2013). Ekdahl et al. found that some of these multicellular phenotypes, namely the flor and biofilm formation, correlated with plastic adherence. However, pseudohyphae formation was observed even when the fungi displayed low levels of adherence, indicating that it developed independently of this trait and was instead a result of nutrient limitation.

To investigate how these multicellular adaptations impact how well the fungus can cause disease, Ekdahl et al. infected wax moth larvae with *S. cerevisiae*. As well as allowing relatively rapid screening of a large number of microbes at once, results from this insect model often correspond with infections in mice (Smith and Casadevall, 2021). Interestingly, the findings showed that fungi that had adapted to display multicellularity also showed increased virulence compared to the original strains and adapted isolates that were non-multicellular. This serves as a striking proof of principle that non-living environmental factors, such as plastic surfaces, can coincidentally enhance the pathogenic potential of a microbe.

The work of Ekdahl et al. illustrates several important concepts and has implications for future work on fungal pathogens. First, the findings support the accidental virulence theory by showing that adaptation to an environmental factor, independent of a host, can incidentally increase a microbe’s ability to cause disease. Second, while the genetic basis of the increased multicellularity was not elucidated, the fungal specimens that were generated can be studied in the future to fully understand how the fungi gained these new characteristics. Lastly, showing that plastic adhesion can enhance fungal virulence serves as a warning as environmental pollution with microplastics becomes increasingly ubiquitous (Hale et al., 2020). Consequently, environmental fungi may become better adapted to living on plastic surfaces, resulting in them gaining virulent traits which could potentially contribute to emergence of new types of fungal infection.
